# Associations between Symptoms and Exercise Barriers in Breast Cancer Survivors

**DOI:** 10.3390/jcm12206531

**Published:** 2023-10-14

**Authors:** Hunter Scott, Nashira I. Brown, Erica A. Schleicher, Robert A. Oster, Edward McAuley, Kerry S. Courneya, Philip Anton, Diane K. Ehlers, Siobhan M. Phillips, Laura Q. Rogers

**Affiliations:** 1 Heersink School of Medicine, University of Alabama at Birmingham, Birmingham, AL 35233, USA; 2 Department of Health Behavior, School of Public Health, University of Alabama at Birmingham, Birmingham, AL 35233, USA; 3 O’Neal Comprehensive Cancer Center, University of Alabama at Birmingham, Birmingham, AL 35233, USA; 4 Division of Preventive Medicine, Department of Medicine, University of Alabama at Birmingham, Birmingham, AL 35233, USA; 5 Department of Kinesiology and Community Health, University of Illinois at Urbana-Champaign, Urbana, IL 61820, USA; 6 The Cancer Center at Illinois, Urbana, IL 60632, USA; 7 Faculty of Kinesiology, Sport, and Recreation, University of Alberta, Edmonton, AB T6G 2R3, Canada; 8 School of Human Sciences, Southern Illinois University Carbondale, Carbondale, IL 62910, USA; 9 Department of Quantitative Health Sciences, Mayo Clinic, Phoenix, AZ 85054, USA; 10 Department of Preventive Medicine, Northwestern University Feinberg School of Medicine, Chicago, IL 60611, USA

**Keywords:** exercise barriers, oncology, breast cancer, survivorship, exercise counseling, cancer-related fatigue

## Abstract

Despite exercise benefits for cancer survivor health, most breast cancer survivors do not meet exercise recommendations. Few studies have examined associations between psychosocial symptoms and exercise barriers in this population. To improve physician exercise counseling by identifying survivors with high barriers in a clinical setting, associations between breast cancer symptoms (fatigue, mood, sleep quality) and exercise barriers were investigated. Physically inactive survivors (*N* = 320; average age 55 ± 8 years, 81% White, 77% cancer stage I or II) completed a baseline survey for a randomized physical activity trial and secondary analyses were performed. Potential covariates, exercise barriers interference score, Fatigue Symptom Inventory, Hospital Anxiety and Depression Scale (HADS), and Pittsburgh Sleep Quality Index were assessed. Based on multiple linear regression analyses, only HADS Global (B = 0.463, *p* < 0.001) and number of comorbidities (B = 0.992, *p* = 0.01) were independently associated with total exercise barriers interference score, explaining 8.8% of the variance (R^2^ = 0.088, F(2,317) = 15.286, *p* < 0.001). The most frequent barriers to exercise for survivors above the HADS clinically important cut point included procrastination, routine, and self-discipline. These results indicate greater anxiety levels, depression levels, and comorbidities may be independently associated with specific exercise barriers. Health professionals should consider mood and comorbidities when evaluating survivors for exercise barriers, and tailoring exercise counseling.

## 1. Introduction

Breast cancer is the most diagnosed cancer among women [1]. Over 3.6 million U.S. women are living with a history of breast cancer [2]. Although incidence of newly diagnosed breast cancer is slowly rising each year, the five-year relative survival rate is 90% because most women are diagnosed prior to advanced cancer progression [3]. Engaging in regular exercise is one of the most cost-effective and beneficial interventions available for improving health [4]. It is especially important after a history of breast cancer because breast cancer survivors participating in regular physical activity have a lower risk of cancer-related and all-cause mortality than inactive survivors [5]. However, most breast cancer survivors fail to meet exercise recommendations [6]. Exercise also improves treatment-related symptoms experienced by survivors, including reducing fatigue, depression, and anxiety, while improving perceived sleep quality and quality of life [7,8]. Despite the multitude of publications examining the psychosocial determinants of exercise behavior in breast cancer survivors, little prior research has specifically studied associations between treatment-related symptoms and perceived barriers to exercise in survivors [9,10].

Fatigue, depression, anxiety, and sleep disturbances are commonly reported by survivors, with fatigue being one of the most persistent symptoms [11,12]. Studies indicate that between 70–100% of cancer survivors experience “unrelenting” fatigue often described as “a complete shutdown” [9,13,14]. An overall depression prevalence of 39.9% has been estimated, with 21.2% of breast cancer survivors experiencing “severe” symptoms [15]. It has been suggested that many breast cancer survivors’ depressive symptoms do not reach the clinical threshold for a diagnosis [16]. Although less prevalent than depression, over one quarter (27.2%) of breast cancer survivors experience anxiety [15]. Additionally, breast cancer survivors often report difficulty sleeping at rates twice as high as those without a history of cancer [17]. Estimates of the prevalence of sleep disturbances vary widely between 12–95%.

### 1.1. Connection to Patient Care

Perceived barriers are a common reason why many people do not engage in exercise [18,19]. In a previous study, approximately 73% of breast cancer survivors perceived at least one individual-level barrier to engaging in physical activity [20]. Exercise barriers and treatment-related symptoms often intersect. For example, among cancer survivors, the vast majority report fatigue (78%) as the most common barrier. Trouble getting motivated and staying disciplined were additional exercise barriers reported by survivors [21]. These data warrant further investigation to better understand the relationship between treatment-related symptoms and perceived barriers to physical activity.

If health care professionals have more information regarding the factors impeding exercise behavior in breast cancer survivors, they can be more successful in counseling the large number of survivors who need to begin an exercise routine. Physicians play an invaluable role in exercise promotion since evidence supports counseling patients in the primary care setting to increase exercise [22]. In breast cancer survivors specifically, oncologist recommendations for physical activity results in greater reported exercise [23].

Despite research affirming physician exercise counseling as an effective intervention, patients often leave the clinic without this valuable information. Only one in three patients reported discussing physical activity with their physician in the past year [22]. Physicians frequently report time as the leading reason to avoid exercise counseling, despite several meta-analyses that showed counseling by primary care physicians, even as short as three to five minutes, increases patient physical activity [22]. Hence, a better understanding of the relationship between symptoms and exercise barriers could help physicians more efficiently prepare for and implement brief exercise counseling for breast cancer survivors with fatigue, depression, anxiety, or poor sleep quality.

### 1.2. Study Objectives

The objective of this study was to determine the associations between treatment-related symptoms (fatigue, depression, anxiety, sleep quality) and perceived exercise barriers. Our hypothesis is that breast cancer survivors with the most fatigue, anxiety, depression, and sleep dysfunction, will have the highest reported barriers to exercise, with the most frequent barriers being physical or motivation related. This study has potential to improve health professional exercise counseling and related promotion programs for survivors, by providing practitioners additional information to help tailor exercise prescription to these treatment-related symptoms.

## 2. Methods

### 2.1. Study Design

A secondary data analysis was conducted on 320 breast cancer survivors who completed the baseline survey for a multi-center randomized controlled exercise behavior change trial (R01CA136859 entitled Better Exercise Adherence after Treatment for Cancer [BEAT Cancer] and its U01CA136859 supplement entitled Comparing doubly labeled water to accelerometer to assess physical activity measurement error during and after a physical activity behavior change intervention [COMPARE]) [24]. The study was approved by the University of Alabama at Birmingham, University of Illinois at Urbana-Champaign, and Southern Illinois University School of Medicine Institutional Review Boards for Human Subjects. Selection of participants was previously described in detail [24,25]. Both trials had the same criteria for inclusion and were combined because COMPARE was a result of a grant supplement that funded extension of the original trial infrastructure and procedures. Participants were recruited through cancer support groups, flyers, and newspaper advertisements. English speaking women between 18 and 70 years of age with a history of ductal carcinoma in situ (DCIS) or Stage I, II, or IIIA breast cancer who were post-primary cancer treatment met eligibility criteria. Participants had to be medically cleared by their physician and must have reported engaging in no more than 60 min per week of moderate intensity activity or 30 min per week of vigorous intensity physical activity on average in the previous 6 months.

### 2.2. Measures

A cross-sectional analysis of the survey data collected at baseline was performed. These data included self-reported demographic (e.g., age, race, ethnicity, annual household income, and education), medical (e.g., comorbidities), and cancer (e.g., stage, months since diagnosis, and treatment type) factors. Number of comorbidities was assessed using the 18-item Functional Comorbidity Index [26]. Medical conditions included bone/joint changes; respiratory diseases; cardiovascular disease; neurological conditions; stroke; diabetes mellitus; gastrointestinal disease; hearing or visual impairment; obesity; and mood disorders. Body mass index (BMI) was measured in person by trained research staff using a scale and stadiometer [calculated using weight (kg)/height (m^2^)].

Participants’ perceived barriers to exercise were measured by a 5-point Likert scale (1 = never to 5 = very often) on how often 21 different barriers (pain, cost, time, fatigue, etc.) interfere with exercise [27]. Frequency of barriers interference was analyzed by each individual barrier and then was summed for an overall exercise barrier score. A higher score is indicative of more perceived barrier interference to exercising.

Fatigue was measured using the 13-item Fatigue Symptom Inventory (FSI) with a higher value indicating greater fatigue [28]. Fatigue interference (mean of 7 items) and fatigue intensity (mean of 4 items) were assessed with 10-point Likert scale options (1 = not at all fatigued to 10 = as fatigued as I could be). A mean score ≥ 3 has been suggested as a cut point optimizing sensitivity and specificity for detecting clinically significant fatigue [29].

Anxiety and depression were measured using the 14-item Hospital Anxiety and Depression Scale (HADS). A higher value indicates more anxiety and depressive symptomatology [30]. A cut point of ≥16 for clinically important anxiety or depression is frequently used [31]. 

Sleep dysfunction was assessed using the Pittsburgh Sleep Quality Index (PSQI), which uses 19 individual items to generate a global score for sleep dysfunction over the past month. A higher score indicates worse sleep, with a cut point of 5 differentiating good vs. poor sleep [32]. 

### 2.3. Data Analysis

Pearson correlation coefficients (*r*) were calculated to determine if participants with higher levels of fatigue, anxiety, depression, and sleep dysfunction, also have the highest overall perceived barriers to exercise. Next, potential covariates were identified by calculating Pearson correlation coefficients between symptoms and age, cancer stage, months since diagnosis, number of comorbidities, and BMI. To assess the association of each symptom by race (White vs. non-White), income (≤$49,999 vs. ≥$50,000), marital status (married or living with significant other vs. single), education (≤12 vs. >12 years), history of chemotherapy (yes/no), radiation treatment (yes/no) or hormonal therapy (yes/no), independent t-tests were performed. A similar approach was used to assess associations between potential covariates and exercise barriers score. Based on these results, a partial correlation including each symptom and exercise barrier score, adjusted for comorbidity score (only covariate identified), was executed. Using the results of these correlations to identify covariates of the symptoms (independent of each other), a multiple linear regression was performed including all symptoms and number of comorbidities. Once HADS was identified as associated with barriers independent of the other symptoms, post hoc exploratory analyses comparing the prevalence of individual barriers above and below the HADS cut point of ≥16 for clinically important anxiety or depression were completed [31]. All statistical procedures were completed using IBM SPSS Statistics 28.

## 3. Results

### 3.1. Participants

The mean participant age was 55 ± 8 years with a mean number of comorbidities of 2.2 ± 1.8 and mean BMI of 31.1 ± 7.34. Most participants were White (81%), married/living with significant other (69%), and had received an Associate Degree or higher (72%). Most participants had a history of stage I (39%) or stage II (38%) cancer. The mean time since diagnosis was 53 months. Treatment varied, with half of participants reporting current hormonal therapy, 66% reporting prior radiation treatment, and 62% stating they had prior chemotherapy. Participants self-identified as having the following medical comorbidities: arthritis (rheumatoid and/or osteoarthritis) (31%); osteoporosis (9%); asthma (16%); chronic obstructive pulmonary disease, acquired respiratory distress syndrome, or emphysema (2%); angina (1%); heart attack (myocardial infarct) (0%); neurological disease (e.g., multiple sclerosis or Parkinson’s) (1%); stroke or TIA (3%); peripheral vascular disease (2%); diabetes mellitus type I or II (10%); upper gastrointestinal disease (e.g., ulcer, hiatal hernia, reflux) (27%); depression (32%); anxiety or panic disorders (17%); visual impairment (e.g., glaucoma, macular degeneration) (10%); hearing impairment (very hard of hearing) (3%); degenerative disc disease (e.g., spinal stenosis, or chronic back pain) (13%); and obesity (40%). Table 1 displays further information.

### 3.2. Identification of Covariates

Number of comorbidities showed a statistically significant correlation with exercise barriers score (*r* = 0.197, *p* < 0.001), HADS Global Score (*r* = 0.239, *p* < 0.001), PSQI Global Score (*r* = 0.157, *p* = 0.005), fatigue mean intensity score (*r* = 0.184, *p* < 0.001), and fatigue mean interference score (*r* = 0.323, *p* < 0.001). Only number of comorbidities was statistically significantly associated with both symptoms and barriers score.

### 3.3. Adjusted Correlations between Symptoms and Exercise Barriers

Table 2 shows descriptive statistics and a partial correlation matrix of symptoms and total exercise barriers adjusted for number of comorbidities. The most relevant statistically significant correlations with exercise barrier score, adjusted for number of comorbidities, included HADS Global Score (*r* = 0.23, *p* < 0.001), fatigue mean interference score (*r* = 0.17, *p* < 0.01), and PSQI Global Score (*r* = 0.19, *p* < 0.001). There was a statistically significant difference (*p* < 0.001) in exercise barriers score between survivors below (M = 56.8, SD = 12.4) and above (M = 62.6, SD = 12.4) the HADS cutoff, *t*(318) = −3.668, *p* < 0.001.

### 3.4. Linear Regression

A multiple linear regression was performed to determine which symptoms are independently associated with exercise barrier score. Previously determined covariates (i.e., number of comorbidities) and symptoms were used in the linear regression. Fatigue mean interference score was the fatigue measure selected for inclusion in the model because it had the highest unadjusted correlation with exercise barrier score. A stepwise multiple linear regression was calculated to predict exercise barrier score based on number of comorbidities, HADS Global Score, PSQI Global Score, and fatigue mean interference score. This was performed in order to develop the most parsimonious model for predicting exercise barriers score based on the variables that were significantly correlated with this outcome (PSQI, fatigue mean interference scores, HADS Global, and number of comorbidities). In this stepwise regression model, the probability of F to remove was set to 0.05. The results of the final stepwise linear regression are displayed in Table 3. PSQI Global Score and fatigue mean interference score did not reach significance in this model. A significant regression equation was found (F(2,317) = 15.286, *p* < 0.001), with an R^2^ of 0.088. Both HADS Global Score (*p* < 0.001) and number of comorbidities (*p* = 0.01) were significantly associated with exercise barrier score. Backward linear regression model conducted to confirm the results of the stepwise model yielded the same findings.

### 3.5. Prevalence of Individual Barriers above the HADS Clinically Important Cut Point

**Figure 1:**
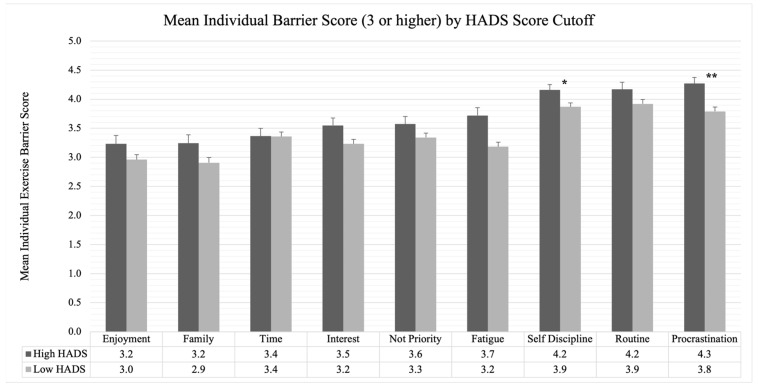
Bar graph of 9 individual exercise barriers with a mean score of 3 or higher separated by breast cancer survivors with high HADS score and low HADS score above and below the HADS cut point of ≥16 for clinically important anxiety or depression [32]. Error bars indicate standard error. Independent samples t-test demonstrated a statistically significant difference between survivors with high and low HADS scores on self-discipline (*p* = 0.019) and procrastination (*p* < 0.001). * *p* < 0.05. ** *p* < 0.001.

To identify the most common barriers faced by breast cancer survivors, the mean individual exercise barriers score of each of the twenty-one perceived barriers was calculated and the most frequent barriers identified. Figure 1 displays the most common barriers of participants above and below the HADS cutoff. Only individual barriers with a mean score of three (“Sometimes”) or higher are shown in Figure 1. There was a statistically significant difference (*p* < 0.001) in exercise barriers score between survivors below (M = 56.8, SD = 12.4) and above (M = 62.6, SD = 12.4) the HADS cutoff, *t*(318) = −3.668, *p* < 0.001. Based on a post hoc analyses of individual barriers, the most frequent barriers for survivors above the HADS cut point for clinically important anxiety or depression included procrastination (M = 4.27, SD = 0.91), routine (M = 4.17, SD = 1.11), and self-discipline (M = 4.16, SD = 0.84). Evaluation of barriers in survivors below the HADS cut point revealed the same barriers: procrastination (M = 3.79, SD = 1.19), routine (M = 3.92, SD = 1.14), and self-discipline (M = 3.87, SD = 0.99). Independent samples t-tests were performed to generate hypotheses related to the differences between survivors with noteworthy anxiety and depression by individual barriers. Survivors with clinically important HADS scores reported more barriers related to self-discipline (*p* = 0.019) and procrastination (*p* < 0.001) compared with survivors below the clinical cut point.

## 4. Discussion

The results of the study indicate that levels of anxiety and depression, sleep dysfunction, and fatigue are significantly correlated with perceived exercise barrier score. Contrary to other research which typically has focused on fatigue, our analysis showed that mood, compared with fatigue, may be more strongly correlated with higher perceived exercise barriers, especially self-discipline and procrastination barriers [9,33,34]. Linear regression models showed that the number of comorbidities and HADS score were independently associated with exercise barrier score. Exploratory analyses of individual barriers by mood (above and below the clinically important cut point) suggest that the most frequent barriers were motivation related (i.e., self-discipline and procrastination) and no single barrier was vastly different between survivors with high or low HADS scores. Also, all barriers, except weather, were the same or higher in the high anxiety/depression group generating useful hypotheses warranting further research in larger sample sizes. 

Few prior studies have investigated possible associations between psychosocial symptoms and exercise barriers in breast cancer survivors. One qualitative study of cancer survivors with prominent fatigue supports the premise that these patients have treatment-related exercise barriers, usually originating from fatigue. The study was limited by its enrollment of only twelve participants (all Caucasian) [9]. Only one paper was located that examined barriers by level of fatigue and depression. While this study (*N* = 162) supports the premise that survivors with high barriers to physical activity were more likely to experience high levels of depression and fatigue, no prior study comparing barriers by level of anxiety was located. The study was again limited by its participants’ income composition [10]. 

Our study supports findings from prior research that found higher levels of fatigue and depression were associated with exercise barriers [10]. The previously mentioned study found ‘‘Lack of time’’, “Lack of self-discipline”, and ‘‘Fatigue (or lack of energy)’’ to be the barriers to exercise with the highest mean scores [10]. Our results reiterate lack of self-discipline and fatigue are common perceived barriers across various levels of anxiety and depression; however, we did not find lack of time to be a significant contributor. The results of a linear regression in the above study found age, ethnicity, marital status, education, and treatment history to be non-significant, as was found in our independent groups t-tests. In contrast to these results, our study found number of comorbidities to be significantly associated with barrier score. BMI did not have a statistically significant association with the symptoms. Furthermore, we determined anxiety and depression to be significantly associated with barrier score independent of fatigue interference. The previous study did not evaluate anxiety or sleep quality [10].

Health care professionals can reduce survivors’ anxiety and depression using exercise prescription, but they must consider the relationship between mood and perceived exercise barriers to effectively assist their patients in adhering to the exercise prescribed. The results of our study generate more awareness of the potential higher exercise barriers in breast cancer survivors with mood dysfunction. Thus, physicians might make discussions with survivors about their mood a regular hallmark of every visit. Insufficiently physically active survivors with anxiety or depression may feel like a failure regarding their exercise habits and are probably unaware that they are more likely to face exercise barriers [35]. 

Our study has the potential to aid in shifting conversation from simply stating exercise recommendations, to instead emphasizing the importance of physicians targeting the exercise barriers faced by survivors to therefore promote life-long physical activity. One major limitation on the part of physicians attempting to deliver exercise counseling is their limited time [36]. To provide support in addressing this challenge, our study demonstrates initial signals that the identification of survivors with high HADS scores and extensive comorbidities may be used by providers to engage in more extensive conversations with patients regarding their specific barriers and potential coping mechanisms. Further, physicians armed with more information about the possible relationship between barriers, mood disturbance, and comorbidities might be able to suggest better medical optimizations or assist in making more social services available to survivors with the goal of alleviating their barrier burden. Beyond oncologists, the study results are applicable to psychologists and psychiatrists treating breast cancer survivors. Treating the underlying mood disorder in survivors may be needed before the individual has the emotional reserve to consider an exercise routine. While the converse must also be stated that survivors may experience reductions in mood disturbances with regular exercise, as demonstrated by reductions in anxiety and depressive symptoms reported after 12 weeks of thrice weekly aerobic exercise [7]. Because of the strong evidence supporting the beneficial effects of exercise on mood as documented in a recent consensus statement by the American College of Sports Medicine, it is not surprising that the American Society of Clinical Oncology guidelines for treatment of mood disorders in cancer survivors include exercise as a non-pharmacologic treatment modality [7,37]. Psychiatrists for example might consider using screening tools, like the HADS, in identifying breast cancer survivors with the highest barriers to exercise to better encourage and support barriers coping and resultant engagement in regular aerobic exercise [4].

### 4.1. Strengths and Limitations

Our study’s relatively large number of participants (*N* = 320) is a key strength. We were very selective about the variables used based on our a priori study objectives thus, limiting the number of comparisons. However, the associations were of sufficient strength to remain statistically significant even if multiple comparisons (such as the Bonferroni Correction) were done. The symptoms included in our findings often cluster in survivors, thus determining the treatment-related symptoms (i.e., HADS) independently associated with barriers can better guide clinical practice. While our study included more African American survivors (15%) than other studies (5.6% in Ventura et al., 0% in Blaney et al.), and met census criteria for African American participant inclusion based on the study location, the study’s generalizability to Hispanic and Latino women is limited due to a low number of participants (2%) identifying as such. It is desirable that future research be conducted to expand upon the unique barriers faced by African American and Hispanic/Latino survivors, along with those of other racial and ethnic minorities [9,10]. Our inclusion of anxiety and depression, as well as sleep quality, in the analysis is noteworthy since most previous literature has centered on fatigue and determining the specific exercise barriers faced by breast cancer survivors. Questions remain regarding associations between socioeconomic status and exercise barriers, as race, income, and education were not significantly associated with barriers in our analysis possibly due to limited variability in these covariates. Compared to previously published research, our analysis fills the knowledge gap surrounding exercise barriers and level of depression, anxiety, and sleep quality, and includes more participants across a wider range of ages.

### 4.2. Implications

Because a significant proportion of breast cancer survivors struggle with anxiety, depression, fatigue, and disordered sleep following their diagnoses, determining possible associations between symptoms and exercise barrier interference empowers physicians to better counsel survivors on regular exercise. Moreover, survivors with the highest levels of fatigue, depression, anxiety, and sleep dysfunction have the most to gain from regular exercise [38].

Although prior studies have reported an association between fatigue and exercise barriers, further research elucidating the role of mood in exercise barriers among breast cancer survivors is needed [9,10,34,39]. Additional research should evaluate anxiety, depression, and sleep quality in breast cancer survivors because prior study has emphasized fatigue as a key factor in exercise barriers, yet fatigue inference was not significant independent of mood in our linear regression. Number of comorbidities should also be examined since our results indicate it is strongly associated with barriers. 

Health professionals should consider screening for mood and assessing comorbidities when evaluating breast cancer survivors for exercise barriers and tailoring exercise counseling. A rising proportion of oncology practitioners across continents are integrating assessment of treatment-related symptoms into their patient care, with nearly 25% of professionals reporting that they conduct symptom assessments on a vast majority (>80%) of patients [40]. Considering the results of our study, survivors with high levels of anxiety and depression, along with medical comorbidities, will likely benefit from additional time spent with their physician on exercise counseling and probable higher perceived barriers to exercise. The growing access to symptom assessments as part of routine clinical care further enhances the relevance and application of our study results.

## Figures and Tables

**Table 1:** Female Breast Cancer Survivors (*N* = 320).

Variable	Breast Cancer Survivors ^a^
Age (years)	55 ± 8 (21–70)
Ethnicity	
Not Hispanic/Latino	315 (98)
Missing	1 (0.3)
Race ^b^	
White/Caucasian	260 (81)
Black/African American	48 (15)
Asian	8 (3)
Native American/Alaska Native	3 (1)
Native Hawaiian/Pacific Islander	2 (1)
other	4 (1)
Income	
<$10,000	14 (4)
$10,000 to $19,999	16 (5)
$20,000 to $34,999	33 (10)
$35,000 to $49,999	39 (12)
$50,000+	214 (67)
Missing	4 (1)
Education	
some high school	2 (0.6)
high school	57 (17.8)
some college	32 (10)
associate degree	43 (13.4)
bachelor’s degree	82 (25.6)
graduate degree or higher	104 (32.5)
Mean number of comorbidities	2.2 ± 1.8 (0–7)
Mean Body Mass Index (BMI)	31.1 ± 7.34
Marital status	
married or living with significant other	221 (69)
other	99 (31)
Cancer stage	
Ductal Carcinoma in Situ (DCIS)	41 (13)
I	125 (39)
II	121 (38)
III	33 (10)
Estrogen Receptor (self-report)	
Positive	194 (61)
Negative	61 (19)
Unsure	62 (19)
Missing	3 (1)
Months since cancer diagnosis	53 ± 54 (2–276)
Current hormonal therapy (yes)	160 (50)
Prior radiation therapy (yes)	210 (66)
Prior chemotherapy (yes)	197 (62)

^a^ Values presented as mean ± standard deviation (range) or number (percent). ^b^ Participants may select multiple races.

**Table 2:** Descriptive Statistics and Partial Correlation Matrix of Symptoms Adjusted for Number of Comorbidities (*N* = 320).

Variable	M	SD	1	2	3	4	5
1. HADS Global	11.5	6.3	–				
2. PSQI Global	8.2	4.1	0.46 ***	–			
3. fatigue mean interference	3.4	2.1	0.51 ***	0.46 ***	–		
4. fatigue mean intensity	4.5	1.9	0.40 ***	0.40 ***	0.74 ***	–	
5. exercise barriers	58.3	12.7	0.23 ***	0.19 ***	0.17 **	0.17 **	–

*** *p* < 0.001. ** *p* < 0.01. Abbreviations: HADS Global, Hospital Anxiety and Depression Scale Global Score; PSQI Global, Pittsburgh Sleep Quality Index Global Score.

**Table 3:** Summary of Linear Regression for Variables Associated with Barrier Score.

Variable	B	SE B	β
**HADS Global**	0.463	0.112	0.228 **
**Comorbidities**	0.992	0.385	0.142 *
**R^2^**	0.088		
**F**	15.286 **		

* *p* < 0.05. ** *p* < 0.001. Abbreviations: B, unstandardized beta coefficient; SE B, standard error unstandardized beta; β, standardized beta coefficient; Comorbidities, Comorbidity Score; HADS Global, Hospital Anxiety and Depression Scale Global Score.

## Data Availability

Data available upon request.
